# Dose-Response Modeling with Summary Data from Developmental Toxicity Studies

**DOI:** 10.1111/risa.12667

**Published:** 2016-08-27

**Authors:** John F. Fox, Karen A. Hogan, Allen Davis

**Affiliations:** National Center for Environmental Assessment, U.S. EPA, Washington, DC.

**Keywords:** Benchmark dose, design effect, developmental, dose response, fetal, intralitter correlation, overdispersion

## Abstract

Dose-response analysis of binary developmental data (e.g., implant loss, fetal abnormalities) is best done using individual fetus data (identified to litter) or litter-specific statistics such as number of offspring per litter and proportion abnormal. However, such data are not often available to risk assessors. Scientific articles usually present only dose-group summaries for the number or average proportion abnormal and the total number of fetuses. Without litter-specific data, it is not possible to estimate variances correctly (often characterized as a problem of overdispersion, intralitter correlation, or “litter effect”). However, it is possible to use group summary data when the design effect has been estimated for each dose group. Previous studies have demonstrated useful dose-response and trend test analyses based on design effect estimates using litter-specific data from the same study. This simplifies the analysis but does not help when litter-specific data are unavailable. In the present study, we show that summary data on fetal malformations can be adjusted satisfactorily using estimates of the design effect based on historical data. When adjusted data are then analyzed with models designed for binomial responses, the resulting benchmark doses are similar to those obtained from analyzing litter-level data with nested dichotomous models.

## 1. INTRODUCTION

For risk assessment purposes, dose-response analyses can facilitate deriving risk values to support public health standards. The object of these dose-response analyses is to estimate a benchmark dose (BMD), defined as the dose at which a specified increase (benchmark response [BMR]) in adverse response occurs.^(1,2)^

For dose-response analysis of developmental toxicity studies, data from individual litters are generally recommended in order to accurately estimate fetal risk and its variability.^(3–11)^ This is so because fetuses from the same dam are not statistically independent; pups of the same litter tend to respond more alike than do pups from different litters due to similarity in genetics and environment.^(3,4,6–11)^ By using litter-specific data (i.e., counts for each litter), intralitter correlation can be accounted for through such modeling methods as “nested” dichotomous models that rely on a beta-binomial model of variability, or through quasi-likelihood methods, generalized estimating equations, and hierarchical modeling.^(1,6–10,12)^

Unfortunately, data for individual litters are rarely published in peer-reviewed articles or provided in supplementary data. Instead, only dose-group summaries for the number or average proportion abnormal and the total number of fetuses are presented, making it impossible for risk assessors to account accurately for intralitter correlation of binary data in trend tests for hazard evaluations or dose-response modeling for BMD inference.

Therefore, alternative approaches are needed that can use summary statistics of binomial data but still account for intralitter correlation. A body of work has focused on the concept of design effect, introduced in the context of cluster sampling, as a data transformation for reducing overdispersion of binary data. The transformed data can be analyzed by standard methods for independent binomial data, such as trend tests and dose-response analyses.^(5,7,9,12–14)^ Design effect *D* is related approximately to intralitter correlation *ρ*_*I*_ as *D* = [1 + (*n* − 1)*ρ*_*I*_] in the special case that all litters have *n* offspring (in practice, a weighted average litter size is used).^(13)^

In particular, the Rao–Scott approach^(5)^ estimates *D* from litter-specific data, and then divides both the counts of affected offspring and the total counts of offspring by *D*. The derivation and estimation of the Rao–Scott transformation and its application to clustered developmental data were described well by Krewski and Zhu.^(13)^ Briefly, *D* is the ratio of two variances. Both variances require the estimate of proportion affected *P*_*F*_ = *A*_*F*_/*N*_*F*_ for the dose group, where *A*_*F*_ is the number of abnormal offspring and *N*_*F*_ is the number of offspring at risk, disregarding litter membership. The denominator of *D* is the estimated variance of a binomial proportion, *P*_*F*_(1 – *P*_*F*_)/*N*_*F*_, calculated by treating all offspring as independent observations, which underestimates the correct variance. The numerator $\hat{V}$ is an estimate of the correct variance, based on a weighted sum of squared deviations of litter proportions (*p*_*i*_) from *P*_*F*_:

$$\hat{V}=\left(\frac{m}{m-1}\right)\frac{1}{N_{F}}\sum_{i}^{m}n_{i}^{2}{\left(p_{i}-{\hat{P}}_{F}\right)}^{2},$$

where *n*_*i*_ is the number of offspring in the *i*th litter and *m* is the number of litters.^(5)^

The Rao–Scott transformation consists of dividing both the numerator and denominator of a proportion by *D*.^(5,9)^ Division by *D* produces what can be termed *effective* sample sizes *N*_*F*_/*D* and *A*_*F*_/*D*. These transformed data are appropriately handled using models for binomial data. This transformation is an approximation for small *m* and is essentially exact for large *m*^1^. Intralitter correlation, and thus design effect, has been reported to increase with proportion affected,^(6,14)^ motivating the practice of estimating intralitter correlations separately for each dose group.^(3–9,13–15)^

We sought to answer two main questions:

1. Is an analysis using summary data, *adjusted using a Rao–Scott transformation*, sufficiently close to one based on litter-specific data to be adequate for BMD inference? This question was previously examined by simulating samples from a model fitted to one data set,^(9)^ but has not been examined across a range of real data sets exhibiting a variety of dose-response patterns and design effect values. Because the Rao–Scott transformation is an approximation, it is important to verify that the method gives acceptable results for a variety of real data sets.
2. When study-specific estimates of design effects are not available, are approximate adjustments for intralitter correlations, i.e., using design effect values based on historical data, adequate for benchmark dose inference?

These questions were addressed by comparing dose-response analyses of summary data (using models for independent binomial data) to dose-response analyses of litter-specific data using a nested model. A collection of historical developmental toxicology studies was used to estimate design effects. For data reported as counts for each dose group (i.e., total number of live offspring *N*_*F*_ and the number of abnormal live offspring *A*_*F*_), we evaluated a range of values of *D* that might allow satisfactory use of dose-response models for binomially distributed response data. In the absence of direct, study-specific estimates of *D*, alternatives include considering *D* = 1 (i.e., no transformation) and *D* = mean litter size *N*_*F*_/*N*_*L*_, which amounts to using *N*_*L*_ as number at risk and *P*_*F*_•*N*_*L*_ as number affected. Between these two numbers are choices such as using a historical average for *D* and modeling *D* as a function of *P*_*F*_ using historical data.

Another common dose-group summary is the mean and standard deviation of litter proportions of abnormal fetuses, an approach that gives equal weight to each litter. Models intended for continuous, normally distributed data are sometimes applied to these summary statistics; that approach is questionable for the following reasons. For data reported as average litter proportions with standard deviations for each dose group, the normality assumption is clearly violated by data with an underlying binomial distribution. First, this approach relies on the normal approximation for the binomial, which depends on adequate sample sizes (what is adequate increases greatly as proportions approach 0 or 1). Second, litters (indexed *i*) differ in number of offspring (*n*_*i*_), so each observation *p*_*i*_ is generated from a different normal distribution with variance *p*_*i*_ *(1* – *p*_*i*_*)/n*_*i*_. Third, dose-group variance is roughly proportional to a power of the mean but only if all proportions are less than 1/2 (assuming that proportions increase from some small value for controls). Thus, it is not clear how well such data can be modeled using methods intended for normally distributed continuous responses. However, methods suitable for binomial count data should be applicable to such data. The average of litter proportions ($P_{av}=\frac{1}{m}\sum_{i}^{m}p_{i}$), as an alternative estimate of *P*_*F*_ = *A*_*F*_*/N*_*F*_, weights litters equally and might be satisfactory if *P*_*F*_ is not available but *N*_*F*_ is reported. Design effects would need to be considered for estimating effective sample sizes.

Another frequent summary statistic that treats the litter or dam as the experimental unit is the incidence of litters with any abnormal fetuses (incidence of affected litters). Models for dichotomous response data may be applied without an adjustment for design effect. However, it can be shown from elementary probability calculations that the proportion of affected litters will greatly overestimate the proportion of abnormal offspring. In that case, a BMR for fetal abnormalities corresponds to a larger BMR for affected litters. Therefore, several choices of BMR values for affected litters were evaluated.

Only data on fetal malformations are considered here. Although the terms “fetal risk” and “fetal abnormalities” are used, the same principles should apply to any binary outcome measured on fetuses or pups whenever intralitter correlation is present.

## 2. METHODS

### 2.1. Data Sources

Dose-response data were collected for two purposes: (1) estimating design effects with a collection of historical data; (2) comparing dose-response approaches based on summary data, using a subset of studies having an unambiguous dose response. Two data sources were used: (Collection A) the National Toxicology Program (NTP) website and (Collection B) data files originally compiled by Faustman *et al.*:^(16)^

- Nineteen developmental toxicity studies in rodents were acquired, based on availability of an online abstract, a report in the abstract of a significant increase in fetal malformations, and the availability of the individual fetal data. Data on fetal malformations were downloaded from the NTP website. Dose-group totals for litters and fetuses were compared to summaries in the NTP spreadsheets. Incidence of malformations was based on all fetal malformations (external, skeletal, and visceral) in live fetuses (implant code “A”).
- Of the data files compiled by Faustman *et al.*,^(16)^ we used only those identified as NTP or EPA (Environmental Protection Agency) studies. The remaining data sets had fewer doses, fewer test animals, and did not identify tested chemicals. We found 48 files coded “MO” (the code for total malformed fetuses) for mice, rabbits, and rats. Test animal species were identified by codes in the data files; the codes “MI,” “RT,” and “RB” corresponded to mice, rats, and rabbits. Eleven studies (identified in the additional supporting information) duplicated studies from Collection A. Only the latter, downloaded more recently from NTP, were used, leaving 37 studies in Collection B.

The combined database consisted of 55 studies: 21 with mice, 24 with rats, and 10 with rabbits. There were 241 distinct combinations of study and dose group: 94 for mice, 104 for rats, and 43 for rabbits. These 241 dose groups formed the basis for estimating historical design effect averages. For nine dose groups, the proportion malformed (*P*_*F*_) was zero, and these groups were omitted from analyses and plots using log(*P*_*F*_).

For the comparison of dose-response approaches, the combined database was screened to identify studies having a clear response to dose, as demonstrated by:

- A significant trend (*p* < 0.05) using the Cochran–Armitage trend test, adjusted for estimated design effect.^(14)^
- At least a 5% increase in response relative to control, calculated as extra risk, to increase the likelihood of reaching the target BMR of 5% extra risk.

Of studies satisfying these criteria, two were excluded because the response patterns were patently nonmonotonic (percentages malformed were, in order of increasing dosage, 2.3%, 5.8%, 0.5%, 3.8%, and 9.3% for nitrofurazone from Collection B and 2.5%, 1.5%, 7.5%, and 5.4% for bisphenol-a from Collection A). Some of the other data sets chosen for dose-response analysis exhibited minor deviations from monotonicity and single decreases of less than 3% malformed. Thus, 19 data sets were selected for dose-response analysis, 11 from Collection A, and 8 from Collection B. For these studies, numbers of litters ranged from 21–30 for control groups to 7–26 for high-dose groups. Numbers of live fetuses ranged from 173–431 for control groups to 25–285 for high-dose groups.

### 2.2. Data Analysis

After converting all data into R data sets^(17)^ for analysis, the following statistics were calculated for each dose group: number of live fetuses *N*_*F*_, number of malformed live fetuses *A*_*F*_, proportion of malformed live fetuses *P*_*F*_ = *(A*_*F*_*/N*_*F*_), number of litters *N*_*L*_, the number of litters with any malformed fetus, the proportion of litters with any malformed fetus *P*_*L*_ = *(A*_*L*_*/N*_*L*_), the design effect for each dose group (*D*_*g*_), and the mean *P*_*av*_ and standard deviation *S* of the proportion malformed in each litter. Summary statistics for litter size and design effect appear in Table I.

Because estimates of design effect and standard deviation can be highly variable, pooling data within a study is sometimes advisable.^(12)^ Consequently, for each study, we also calculated a single estimate (for all litters ignoring dose group) for the design effect (*D*_*p*_) and calculated a pooled standard deviation of litter proportions (*S*_*p*_) weighted by sample sizes.

**Fig. 1.**
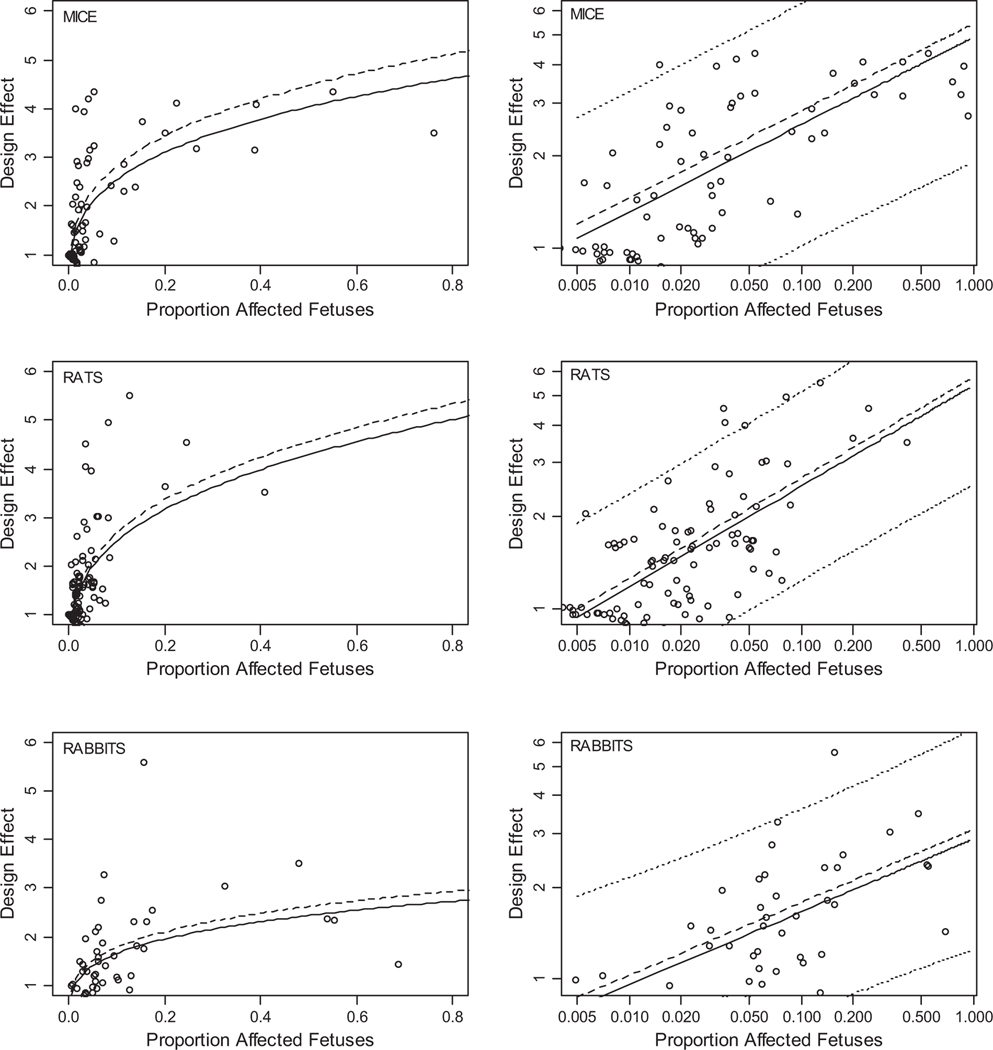
Design effect predicted from proportion malformed fetuses. Lines show predicted mean (dashed), median (solid), and 95% prediction intervals (dotted) in the log-log plots. Based on equations in Table II.

The relationship between dose-group-specific design effects estimated from historical data (*D*_*h*_) and proportions (*P*_*F*_) of affected offspring was estimated using the regression relation log_e_(*D*_*h*_) = *a* + *b* • log_e_(*P*_*F*_), where *D*_*h*_ denotes the predicted design effect. Separate estimates were made for each species (Table II) because rabbits differed significantly and there was a substantial difference between rats and mice. Both *D*_*h*_ and *P*_*F*_ were measured with error, so orthogonal least-squares estimates were compared to least-squares estimates (Table III).^(18,19)^ These coefficients for historical data were used to estimate dose-group-specific design effects (method 3, *D*_*h*_, below), using the average of the orthogonal (OR) and least-squares (LS) estimates for each observation of *P*_*F*_. This average was used because the two methods gave very similar predictions (Table III), and also because LS underestimates slope, while in this case, it is likely that OR overestimates it. Data and least-squares estimates are shown in Fig. 1; there is a significant rank correlation within each species.

### 2.3. Dose-Response Models and Data Transformations

To eliminate or reduce differences owed to model form, models of similar functional form were identified: the nested log-logistic (NLL) model for nested dichotomous data; the log-logistic model for dichotomous data; and the Hill model for continuous data with the asymptote fixed at 1, giving a model with a functional form equivalent to the log-logistic model. Although other software is available, this study used EPA’s BMD software^(20)^ (“BMDS”) because it includes all of the model types identified above, and uses maximum likelihood estimation for parameter estimation and profile likelihood for estimating the BMDL, the 95% lower confidence limit on the BMD. In addition, BMDS is widely used for BMD analyses^(1,2)^ and is accessible to most risk analysts.

We estimated the BMD at a BMR of 5% extra risk, to be consistent with the work of Fung *et al.*^(9)^ Extra risk is defined by *R* = *(P*_*X*_ – *P*_*0*_*)/(1* – *P*_*0*_*)*, where *P*_*X*_ denotes the proportion of animals affected at dose *X*. Thus, *1*–*P*_*0*_ is the proportion of control animals without malformations, and extra risk quantifies the fraction of these that will be affected as dose increases. The BMD and BMDL corresponding to 5% extra risk are inferred from the estimated model.

As a baseline for evaluating transformations of summary data, litter-specific data for the 19 data sets with dose-response trends were modeled using the NLL model. The BMDS NLL uses the beta-binomial probability distribution to account for intralitter correlation (estimating one correlation for each dose group).

For the same 19 data sets, we also examined 15 different combinations of data transformation and model type (“methods”, numbered 1 to 15), to evaluate different ways of modeling the types of summary data described above, organized by type of data summary:

- **Total number of live offspring and number (or percentage) of abnormal offspring**. As noted above, the log-logistic model was selected as the dose-response model for binomially distributed response data. For methods 1–7 below, different values of *D* were applied, via a Rao–Scott-type transformation, to the number of abnormal live offspring *A*_*F*_ and to the total number of live offspring *N*_*F*_ as follows:
  (1) *D*_*g*_, using litter-specific data for each dose group,
  (2) *D*_*p*_, using litter-specific data, pooled across dose groups for the study,
  (3) *D*_*h*_, using species-specific equations (described in Section 2.2) to estimate dose-group- specific design effects from proportions affected *P*_*F*_,
  (4) *D* = 1 (no allowance for intralitter correlation),
  (5) *D* = 2,
  (6) *D* = 3,
  (7) *D* = *N*_*F*_*/N*_*L*_, or mean dose-group litter size (effective *N* equals number of litters *N*_*L*_).
- **Average of litter proportions (*P*_*av*_) and number of offspring (*N*_*F*_).** For methods 8–10, the log-logistic model was applied to the proportion of affected offspring *P*_*av*_ and the number at risk. (BMDS calculates number of affected offspring using the percentage and the effective number at risk, without rounding to whole numbers, thus decreasing rounding error.) For design effects in the Rao–Scott transformation, we used:
  (8) *D* = 1 (paralleling method 4 above),
  (9) *D* = 2 (paralleling method 5 above),
  (10) *D* = *N*_*F*_*/N*_*L*_ (paralleling method 7 above).
- **Average and standard deviation of litter proportions**. For methods 11 and 12, the Hill model for continuous response data (with asymptote fixed at 1) was applied to the averages *P*_*av*_ and the corresponding standard deviations; sample size was the number of litters in the dose group *N*_*L*_. Desgn effects were not used because using dose-group means and standard deviations of litter proportions avoids the litter effect problem; based on a randomized design, litters are assumed to be independent sample units. The following model options were used:
  (11) Hill model with constant variance (*S*_*p*_, pooled variance estimate), “Hill (vc),”
  (12) Hill model with variance modeled as a power of the means, “Hill (vm).”
- **Number of litters and number or proportion of affected litters**. For methods 13–15, the log-logistic model was applied to the percentage of affected litters 100*P*_*L*_ (i.e., litters having at least one abnormal fetus). Sample size was the number of litters in the dose group *N*_*L*_. Design effects were not used because litters are assumed to be independent sample units. BMDs and BMDLs were estimated at 5%, 30%, and 40% extra risk (methods 13–15, respectively).

## 3. RESULTS

Model fit of all 19 data sets was examined graphically for the NLL and for the more successful transformations (methods 1–6) and was seen to be plausible and in no way anomalous. For those models and data, the chi-square goodness of fit was adequate (*p* > 0.10) for 13 to 16 of the 19 data sets (Table IV). Particularly for the baseline NLL fit and methods 1–10, lack of fit based on *p* < 0.10 does not mean that the model deviated greatly from the observations. For method 3 (*D*_*h*_, based on estimating *D* from historical data), predicted and observed *P*_*F*_ differed by less than 50% for 84% of dose groups; the larger relative deviations occurred for smaller *P*_*F*_ and correspond to absolute deviations of 4–5%, though most are less than 3%. As effective sample size decreased (increasing *D*), e.g., methods 4–6, the number of data sets with adequate fit increased; this occurs because lack of fit cannot be detected as readily when effective sample size decreases.

**Fig. 2.**
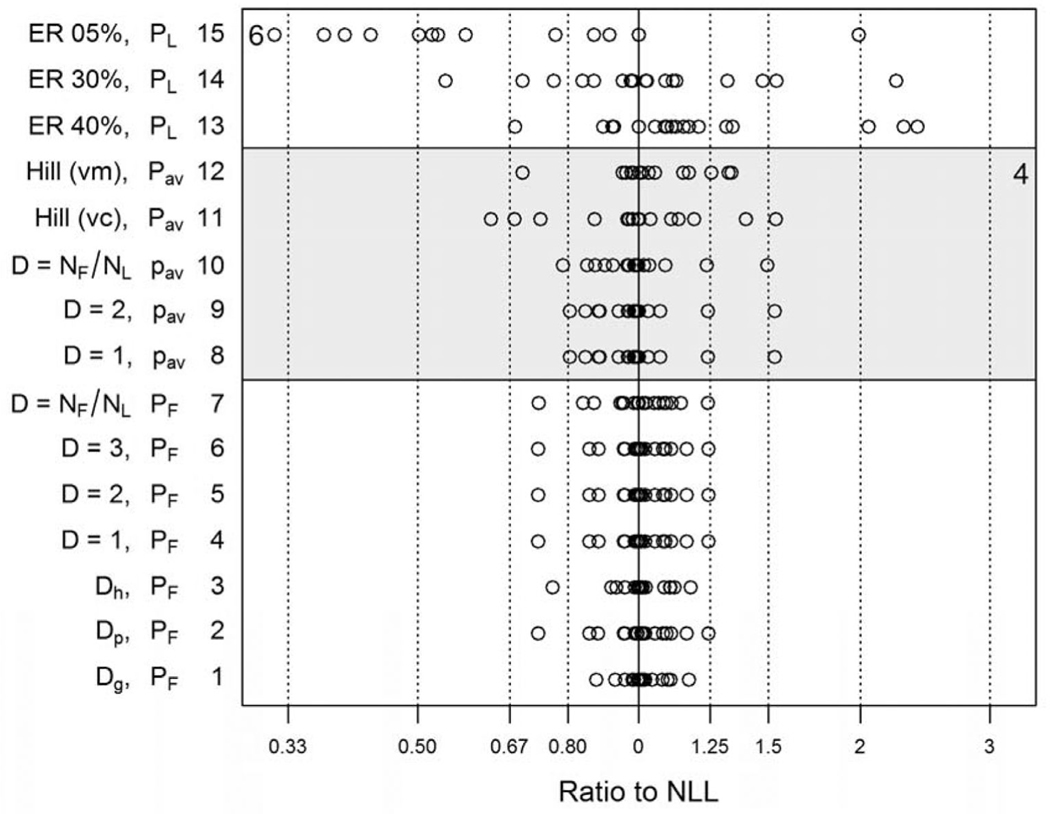
BMDs estimated by various methods using summary data. Nineteen data sets are represented. BMDs are divided by the BMD for the nested log-logistic (NLL) model, and thus expressed as a fraction or multiple of the BMD for NLL. A log_10_ scale is used to represent these ratios and their inverses on an additive scale (e.g., log_10_(2) =−log_10_(1/2) = 0.301). Points to the left of zero represent BMDs lower than that for the NLL model. Numerical notations on the left and right indicate numbers of points off the chart. Also, see the numerical summary in Table IV.

**Fig. 3.**
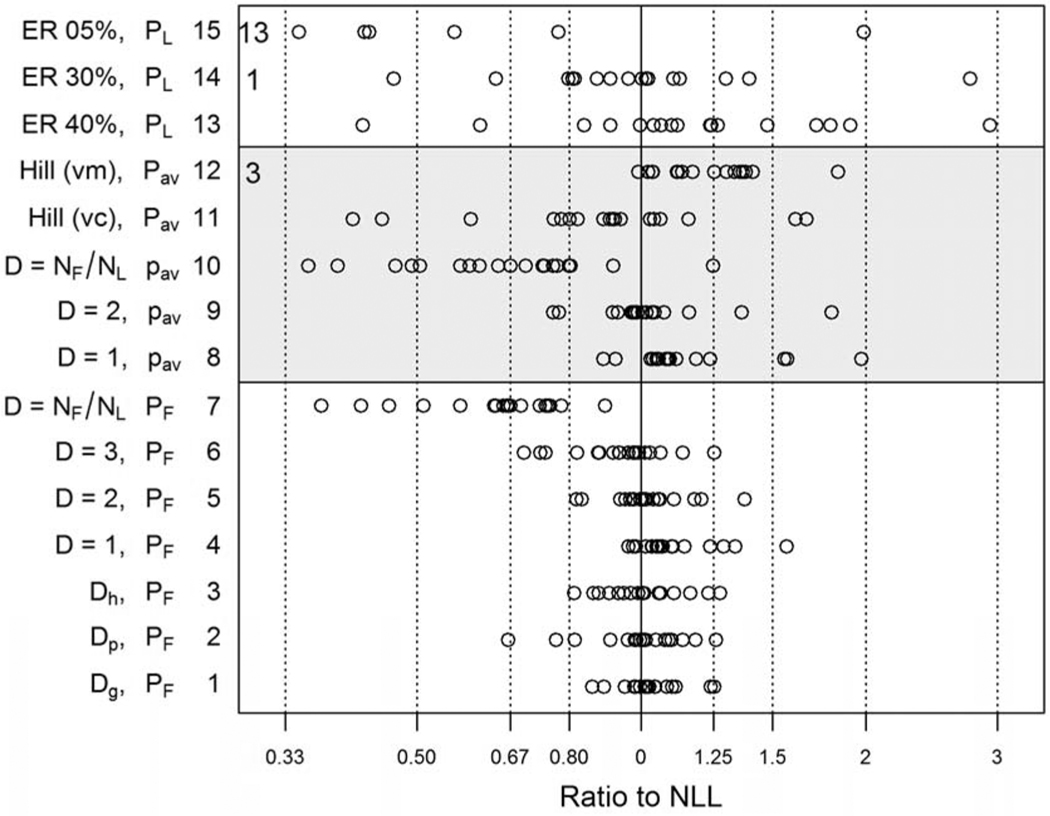
BMDLs estimated by various methods using summary data. Nineteen data sets are represented. BMDLs are divided by the BMDL for the NLL model, and thus expressed as a fraction or multiple of the BMDL for NLL. A log_10_ scale is used to represent these ratios and their inverses on an additive scale (e.g., log_10_(2) = −log_10_(1/2) = 0.301). Points to the left of zero represent BMDLs lower than that for the NLL model. Numerical notations on the left indicate numbers of points off the chart. Also, see the numerical summary in Table IV.

Comparability of the alternative modeling approaches was judged by comparing BMDs and BMDLs to the baseline (NLL) model results. Figs. 2 and 3 compare the BMDs and BMDLs estimated using these different modeling methods with the 19 data sets. The BMD (Fig. 2) or BMDL (Fig. 3) for each model is divided by the corresponding estimate for the NLL model. A log_10_ scale is used to represent these ratios and their inverses on an additive scale (e.g., log_10_(2) =−log_10_(1/2) = 0.301). Summary statistics for the log-ratios are shown in Table IV.

Fig. 2 shows the BMDs as a ratio to the BMD for the NLL model, for all 15 modeling approaches. Methods 1–10 gave BMDs within ±20–30% of the NLL model. Use of a dose- or study-specific design effect (method 1, *D*_*g*_; method 2, *D*_*p*_) gave the least variation from NLL. Four anomalous cases occurred for the continuous Hill model with variance modeled as a power of the mean (method 12), with BMD ratios ≥9.

Fig. 3 shows the BMDLs as a ratio to the BMDL for the NLL model. Use of a dose-specific *D* (method 1, *D*_*g*_) gave BMDLs close to that for the nested model, within ±20–30%. Pooling data over all dose groups in a study (method 2, *D*_*p*_) increased the deviations from NLL slightly and appeared to shift BMDLs slightly lower than those estimated using the NLL model. These two methods are not available when only summary counts are reported, but they provide a benchmark for the other methods. Methods 3–7 correspond to data transformations using *D* estimates external to the data being modeled. Using historical data to predict design effects as a function of P_F_ for each dose group (method 3, *D*_*h*_) was as successful as using *D*_*g*_ (method 1). As expected, using number of fetuses (*N*_*F*_) with no transformation (*D* = 1, method 4) overestimates the effective sample size and tends to give higher BMDLs than does the NLL model. In method 7, using number of litters as effective *N* underestimates effective sample size and gives a smaller BMDL than NLL. Using design effect (*D*) values of 2 or 3 (methods 5 and 6), which are near or slightly higher than averages for these 19 data sets and historical data (Table I), gives intermediate values of BMDL that are centered on or just below the BMDL for NLL, with a range of variation that is only slightly greater than that of the method using design effects estimated from study-specific data (method 1).

The 8th through 12th methods in Figs. 2 and 3 (shaded portion of the figures) apply to data reported as averages of litter-specific proportions abnormal. All of these methods gave more variable results than methods 1–6 based on summaries of count data.

Goodness of fit for the continuous Hill model (methods 11 and 12) was less satisfactory than for the binomial models. When modeling average proportions as continuous response data, the fit of both means and variances is considered. When a constant variance was assumed (method 11), the fit of the Hill model to the means was adequate (BMDS Test 4, *p* ≥ 0.10) for 16/19 data sets (Table IV) but the variance model did not fit well (BMDS Test 3) for any of the 19 data sets. When modeling the variance as a power of the mean (method 12), the means were adequately fitted for only 2/19 data sets, while the variances were fitted well in 13/19 cases (including the two with adequate fit to the means). Also, for method 12, three data sets had extraordinarily small BMDLs (outside the scale of Fig. 3) compared to the BMDL for NLL, and had BMD/BMDL > 100.

The alternative to using a continuous response model when binomial data are summarized as mean percentages of abnormalities is to use the percentage in a dichotomous model (methods 8–10). Different values of effective *N* were tried: number of fetuses *N*_*F*_ (method 8, *D* = 1), *N*_*F*_/2 (method 9, *D* = 2), and number of litters *N*_*L*_ (method 10). Using *N*_*F*_/2 gave BMDLs centered near the BMDL for NLL but with more scatter than the corresponding methods based on count data (methods 4, 5, and 7).

Table IV more precisely quantifies the central tendency and variation visible in Fig. 3, with contributions from bias and variance distinguished. The mean quantifies bias, with a mean of zero indicating ratio of 1. An estimate of root mean squared error (RMSE) was calculated as the square root of the sum of squares of the mean and standard deviation. For reference, if the log-ratios were normally distributed, then in large samples, *σ*_log10_ values of 0.05 and 0.10 imply 95% confidence intervals for the ratio of roughly (0.80, 1.25) and (0.64, 1.57), respectively.

**Fig. 4.**
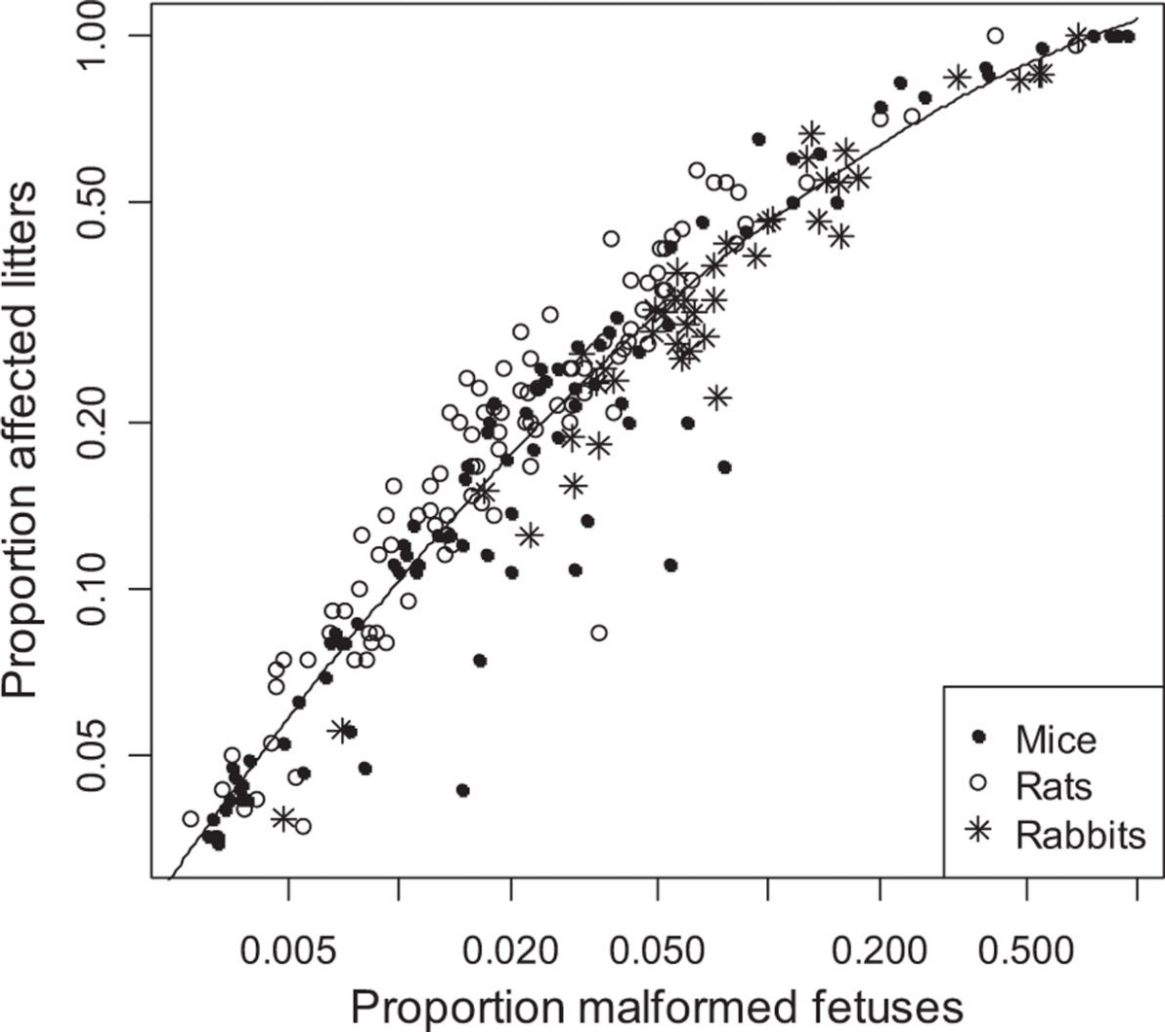
Proportion of litters with a malformed fetus (*P*_*L*_) versus proportion of malformed fetuses (*P*_*F*_) in the dose group, on log_10_ scale. There is one point for each dose group. Nine dose groups with *P*_*F*_ = 0 are omitted. The line is a quadratic fit by ordinary least squares, log(P_L_) = 0.031 +0.226 log(P_F_) −0.141 [log(P_F_)]^2^.

Finally, the relation of proportion of affected fetuses to proportion of affected litters has a direct bearing on the estimation of fetal risk based on a BMD and BMDL from an analysis of affected litters. The relation for the historical data is shown in Fig. 4, where the proportion of affected litters is about 4- to 10-fold greater than proportion of affected offspring. We are interested primarily in *P*_*F*_ ≤ 0.05, and there the relation is essentially log-linear; this relation was quantified using orthogonal least-squares. The model fit was log_10_(*P*_*L*_) = 0.622 + 0.815*log_10_(*P*_*F*_). For *P*_*F*_ = 0.01 and 0.05, this relation predicts that *P*_*L*_ ≈ 0.098 and 0.37. Using ordinary least-squares yields similar estimates, log_10_(*P*_*L*_) = 0.522 + 0.761*log_10_(*P*_*F*_), predicting *P*_*L*_ ≈ 0.10 and 0.34 at *P*_*F*_ = 0.01 and 0.05.

## 4. DISCUSSION

These results illustrate that applying the Rao–Scott transformation to summary data and fitting a model for binomial data provides a reasonably accurate alternative to BMD modeling using a nested model when litter-specific data are not available. Fung *et al.*^(9)^ reached a similar conclusion, for endpoints modeled singly as done here, based on a simulation study using parameters for a single data set. Our report provides a robust confirmation of their conclusion using data for 19 studies. Although litter-specific data are required for estimating design effects, this is a simple calculation. A variety of models and software for binomial data are widely available, thus providing a greater number of model functions that are easier to interpret and report than nested models. Thus, use of the Rao–Scott transformation is a viable alternative to using nested models when litter-specific data are not available.

When only summary data are available, and design effect estimates for those data are not reported, some accuracy and precision for the BMDL, relative to the nested model, are sacrificed. In Fig. 3, methods 1–5 are least variable. *D*_*h*_ and *D* = 2 (methods 3 and 5) seem the best choices for these 19 studies. By applying a design effect of 2 (chosen for simplicity) to all dose groups, results were almost as good as those obtained from using the study- and dose-specific Rao–Scott transformation. The mean design effect for the 19 data sets used for dose-response comparisons was 2.3. However, rather than using a single value for *D,* it is more accurate to estimate design effects for each dose group as a function of estimated proportions (*P*_*F*_) using the relationships reported in Tables II and III, especially when *P*_*F*_ differs greatly among groups. Thus, we recommend method 3 (*D*_*h*_) when litter-specific responses are not available.

By including some data sets for which model goodness of fit did not reach *p* > 0.10, the results are made somewhat conservative, in that the range of BMD and BMDL was likely increased. If a few data sets had been excluded based on model goodness of fit after having been selected based on a significant trend and response of at least 5% above control, this could be regarded as a bias, making the methods appear better (i.e., the range of BMDLs might be narrower) than would occur in practical applications.

Achieving a good model fit was especially difficult when means (*P*_*av*_) and standard deviations of litter proportions were treated as continuous response data. The difficulty of adequately accounting for observed variances is not uncommon and has been addressed insightfully by Slob and Setzer.^(21)^ When variance was modeled (method 12), BMDLs generally exceeded those for the NLL, if the three cases with BMD/BMDL > 10 are excluded as anomalous (these had much smaller BMDLs than did NLL); however, only 2 of the remaining 16 cases achieved adequate fit for both mean and variance. Better results were obtained by using the mean proportion (*P*_*av*_) in a dichotomous response model, with effective sample size *N*_*F*_/2.

It is important to appreciate the relation between risk per offspring and risk per dam or per litter. It follows from elementary probability considerations that the probability of a dam having at least one abnormal offspring is substantially greater than the probability that any one offspring will be abnormal: with a constant litter size (*n*) and independence among fetal responses, *P*_*L*_ = 1 – (1 – *P*_*F*_)^*n*^. Note how *P*_*L*_ depends on litter size. Although independence does not hold because of intralitter correlations, the intralitter correlations are small for *P*_*F*_ < 0.10 (this study and Carr and Portier^(6)^). The relation of proportions of affected litters and fetuses was illustrated by DeClerck *et al.*,^(22)^ using dose-response models fitted to data for several studies: a fetal extra risk of 0.05 corresponded to litter-based extra risks 2- to 8-fold greater. We found that proportion of affected litters is about 4- to 10-fold greater than proportion of affected offspring (Fig. 4). It follows that a much larger BMR must be used in BMD modeling for litters when inference about fetal risk is required and when the only data consist of the proportion of affected litters. Because the relation between *P*_*L*_ and *P*_*F*_ is noisy and effective sample size (*N*_*L*_) is small, it is better to use data on fetal proportions to estimate fetal risk.

The preferred estimator of proportion of fetuses affected is the ratio estimator, *P*_*F*_ = *A*_*F*_*/N*_*F*_.^(5)^ The commonly reported average of litter proportions *P*_*av*_ is slightly larger, by 1–10%, than the ratio estimate, based on our analysis of the historical data. The two estimates would be equal if, in computing the average, litter proportions were weighted by litter sizes.

In the developmental toxicology literature, it is apparently accepted practice^(11,23)^ to summarize data at the litter level (e.g., as proportions of malformations for each litter), and then to summarize those litter values as means and standard deviations for each dose group. This is sensible and efficient if the litter averages are normally distributed with a common variance. However, for binary data, the litter proportions are nonnormal and heterogeneous in variances, so the resulting dose-group summaries (means and standard deviations) cannot be analyzed in the most statistically efficient way because important information has been lost. Another consequence of not reporting data either for each individual offspring or for each litter is that dose-response models for the joint response of two or more outcomes cannot be applied.^(22,24,25)^

The notion that litter effect disqualifies use of any data except that summarized using litter means or litter proportions (e.g., Festing^(23)^) is misguided. Numerous reports (see Section 1) have shown how to analyze developmental data correctly while taking account of intralitter correlation, allowing use of a greater effective sample size. The litter effect does not preclude accurate estimation of fetal risk merely because the dam is the unit of treatment. This article describes methods of dose-response analysis that account for litter effect adequately, using summary data.

When published data are used for BMD modeling without litter-specific data or study-specific design effect estimates, using a design effect predicted as a function of proportion affected, based on estimates from historical data (Tables II and III), is a workable alternative. That approach can be expected to yield BMDLs within about ±50% of a BMDL based on litter-specific data in a nested model. For inference on fetal risk, this degree of imprecision is preferable to the bias of about −50% in the mean, and even greater variation, inherent in using number of litters with percentage of affected fetuses.

We conclude with some recommendations for reporting data from developmental toxicity studies and conducting dose-response analysis with that data.

- First, we encourage authors to report data for each individual offspring, identified as to litter or dam, in a data supplement. If there are two or more outcomes of concern, modeling the joint response can be more protective because it considers overall risk.^(8,9,13,22,24–26)^
- If that level of detail is not possible, litter-specific data by outcome can be sufficient for analysis of single outcomes, although this precludes use of some methods for joint responses.
- If only summary data on numbers of offspring and numbers affected for each dose group will be reported, then design effect values estimated from the data should also be reported. This allows a Rao–Scott transformation to be applied, both for dose-response modeling and for hypothesis testing.^(9,14)^
- Modeling averages of proportions as continuous, normal variates is problematic. We recommend treating the average as a binomial proportion and using the effective number of fetuses, by applying a Rao–Scott transformation, in a model for dichotomous responses.
- If litter-specific data and design effect estimates are not available, estimate group-specific design effects using Tables II or III, divide numbers affected and numbers at risk by design effects, and use models intended for binomially distributed data. Note that our sample sizes for rabbits are smaller and the estimates are less precise.

## Supplementary Material

Supporting Information

## Figures and Tables

**Table I.** Summary Statistics for Design Effects and Average Litter Sizes

	Estimated Design Effect					Average Litter Size				
		
	NTP Downloads		EPA Archived Data			NTP Downloads		EPA Archived Data		
				
Quantiles	Mice	Rats	Mice	Rabbits	Rats	Mice	Rats	Mice	Rabbits	Rats

1	9.8	7.1	7.7	5.6	4.9	13.4	15.8	13.8	8.8	15.2
0.95	4.4	5.1	3.8	3.2	3.7	13.0	15.8	13.2	8.5	14.7
0.90	4.1	3.7	2.9	2.7	2.7	12.5	15.7	12.4	8.2	14.4
0.75	3.2	1.8	2.1	2.2	1.8	11.8	15.4	12.0	7.8	13.6
0.50	1.6	1.4	1.1	1.4	1.4	11.1	14.0	11.4	7.3	13.0
0.25	1.0	1.0	1.0	1.1	1.0	10.6	12.3	11.1	6.8	12.2
0.10	1.0	0.9	0.9	0.9	0.9	8.9	10.1	10.7	6.4	10.9
0.05	0.9	0.8	0.9	0.9	0.9	8.1	9.9	10.4	5.5	10.1
0	0.8	0.8	0.8	0.8	0.8	5.6	7.9	3.6	5.0	8.7
Means	2.3	1.9	1.7	1.7	1.6	10.9	13.5	11.4	7.3	12.8
Dose groups	46	29	48	43	75	46	29	48	43	75
Studies	10	7	11	10	17	10	7	11	10	17

*Notes:* The dose group is the sample unit. Design effect is undefined for nine dose groups having *P*_*F*_ = 0; these dose groups were assigned *D* = 1 for calculations in this table.

**Table II.** Linear Least-Squares (LS) and Orthogonal Regression (OR) Estimates by Species, for the Relation log_e_(*D*) = a + b*log_e_(*P*_*F*_), for Cases with *P*_*F*_ > 0

	Method	Studies	*n*, Dose Groups	*A*	*b*	*σ*_res_^2^

Mice	LS	21	88	1.5938	0.2866	0.2078
Mice	OR	21	88	1.6943	0.3132	0.1863
Rats	LS	25	101	1.6852	0.3310	0.1248
Rats	OR	25	101	1.8327	0.3690	0.1090
Rabbits	LS	10	43	1.0582	0.2397	0.1452
Rabbits	OR	10	43	1.1477	0.2739	0.1299

Estimates of the mean of *D* may be calculated by transforming estimates back from log scale as *D* = exp(*a* + b*log_e_(*P*_*F*_) + 0.5* *σ*_res_^2^).

**Table III.** Estimated Design Effects by Species for a Range of Proportions Malformed, Using Equations of Table II

	*P*_*F*_	0.01	0.05	0.10	0.20	0.30	0.50	0.80

Species	Method							
Mice	LS	1.46	2.31	2.82	3.44	3.87	4.48	5.12
Mice	OR	1.41	2.34	2.90	3.61	4.10	4.81	5.57
Rats	LS	1.25	2.13	2.68	3.37	3.85	4.56	5.33
Rats	OR	1.21	2.19	2.82	3.65	4.23	5.11	6.08
Rabbits	LS	1.03	1.51	1.78	2.11	2.32	2.62	2.94
Rabbits	OR	0.95	1.48	1.79	2.16	2.42	2.78	3.16

**Table IV.** Statistics for log_10_ of Ratios between BMDLs for Various Models (with Data Transformations) and the BMDL for the Nested Log-Logistic Model; the Statistics Summarize Ratios for 19 Data Sets

Data Transformations and Assumptions	Figure 2 ^a^		Source of *N* ^b^	Response ^c^	Fit ^d^	Mean	*SD*	RMSE ^e^

	Fetal risk (BMR 0.05 extra risk). Litter-specific data for each study were used to estimate design effect (*D*.)							
*D* estimated by dose group using study data	1	*N*_*F*_/*D*_*g*_, *P*_*F*_	Fetuses	*P*_*F*_	15	0.012	0.004	0.042
*D* estimated by pooling study data over dose groups	2	*N*_F_/*D*_*p*_, *P*_*F*_	Fetuses	*P*_*F*_	15	−0.005	0.066	0.066
	Fetal risk (BMR 0.05 extra risk). Litter-specific data were not used.							
*D* estimated by dose group using historical data	3	*N*_*F*_/*D*_*h*_, *P*_*F*_	Fetuses	*P*_*F*_	16	0.000	0.051	0.051
*D* = 1	4	*N*_*F*_, *P*_*F*_	Fetuses	*P*_*F*_	13	0.042	0.054	0.068
*D* = 2	5	*N*_*F*_/2, *P*_*F*_	Fetuses	*P*_*F*_	14	0.004	0.056	0.056
*D* = 3	6	*N*_*F*_/3, *P*_*F*_	Fetuses	*P*_*F*_	15	−0.003	0.064	0.071
*D* = *N*_*F*_/*N*_*L*_ (mean litter size)	7	*N*_*L*_, *P*_*F*_	Litters	*P*_*F*_	19	−0.197	0.098	0.221
*D* = 1	8	*N*_*F*_, *P*_*F*_	Fetuses	*P*_*av*_	9	0.058	0.084	0.102
*D* = 2	9	*N*_*F*_/2, *P*_*F*_	Fetuses	*P*_*av*_	14	0.009	0.081	0.081
*D* = *N*_*F*_/*N*_*L*_ (mean litter size)	10	*N*_*L*_, *P*_*F*_	Litters	*P*_*av*_	19	−0.191	0.013	0.231
Fetal risk (BMR 5% increase). Continuous model using mean and SD of litter proportions affected.								
Hill model with variance constant	11	Hill (vc)	Litters	*P*_*av*_	16,0	−0.056	0.151	0.161
Hill model with variance modeled	12	Hill (vm)	Litters	*P*_*av*_	2, 13	−0.026	0.979	1.013
	Litter risk (comparing BMRs for 40%, 30%, and 5% extra risk.)							
Number of litters affected, BMR 0.40 extra risk	13	40% ER	Litters	*P*_*L*_	17	0.065	0.183	0.194
Number of litters affected, BMR 0.30 extra risk	14	30% ER	Litters	*P*_*L*_	17	−0.041	0.199	0.203
Number of litters affected, BMR 0.05 extra risk	15	5% ER	Litters	*P*_*L*_	17	−0.640	0.383	0.746

a Column “Figure 2“ gives order (bottom to top) and notation in Figure 2.

b “Source of *N*” indicates the basis of sample size per dose group before division by design effect (*D*).

c “Response” is the basis of the response variable, before division by design effect; BMDS calculates number affected as (percentage affected)*(effective sample size).

d Fit: number of data sets for which the *p*-value for chi-square goodness of fit exceeded 0.10. For the Hill model, the numbers correspond to fitting the means and the variances, respectively.

e “RMSE” is an estimate of root mean squared error, i.e., the square root of the sum of the squares of the mean and standard deviation. For reference, log_10_(1.1) and log_10_(1.2) are approximately 0.041 and 0.079.

## Footnotes

1 Below, *N*_*L*_ rather than *m* will represent number of litters in a dose group.
