# Supplementary material for: Dose-Response Modeling with Summary Data from Developmental Toxicity Studies
Source: Risk Anal. Author manuscript; Available in PMC 2021 Aug 18. (PMC8372781; doi:10.1111/risa.12667)
Supplement: Supporting Information [file NIHMS1544377-supplement-Supporting_Information.zip › models.docx]

**Models as parameterized in BMDS**

**(note: different symbols are used in in BMDS help files)**

**Model for dichotomous (binary) response data**:

Y is number of malformed, N the number at risk

Y ~ f( π(X,θ) | N ), where f is either binomial or, for the nested log-logistic, beta-binomial

Models for the expected probability, π

Nested log-logistic with covariates

π(X,θ) = g + ω_1_ Z + (1 –ω_1_ Z –g) / [1 + exp(–α –ω_2_ Z –β•log(X) ]

Log-logistic, and Nested log-logistic without covariates

π(X,θ) = g + (1-g) / [1 + exp(–α –β•log(X) ]

**Model for normally-distributed (continuous) response data**

Y ~ N(μ, σ^2^)

Hill model for expected (mean) response (g = intercept at x=0; asymptote as x → ∞ is g+c)

μ = g + c • [ X^n^ ∕ ( k^n^ + X^n^ ) ]

σ^2^ = ν•μ^ρ^ (if variance is constant across doses, ρ = 0)

To re-parameterize, multiply second term on r.h.s. by 1 = X^-n^ / X^-n^ , substitute k^n^ = exp(n•log(k)) and x^n^ = exp(–n•log(x)), and identify n = β and -n*ln(k) = α, so that

μ = g + c • [ 1 ∕ ( 1 + exp(–α –β•log(X) ) ]

finally, if c = (1–g) then the asymptote as x → ∞ is 1

In fact, c was fixed at 1 for BMDS estimation in our analyses. This is a reasonable approximation (to 1-g) if g is small (the largest estimate was 0.058) or if the maximum observed response proportion is not near 1.
