# Supplementary material for: Dose-Response Modeling with Summary Data from Developmental Toxicity Studies
Source: Risk Anal. Author manuscript; Available in PMC 2021 Aug 18. (PMC8372781; doi:10.1111/risa.12667)
Supplement: Supporting Information [file NIHMS1544377-supplement-Supporting_Information.zip › Relation of Fetal to Litter Proportions.docx]

"*The proportion of affected litters will exceed the proportion of affected fetuses. It follows from elementary probability considerations that the probability of a dam having at least one abnormal offspring is substantially greater than the probability that any one offspring will be abnormal."*

If fetal responses were independent and binomially distributed and litter size (*n*) were constant, the relation between the proportion of affected litters, P_L_, and the proportion of affected fetuses, P_F_, would be P_L_ = 1 – (1 – P_F_)*^n^* , or (1- P_L_) = (1 – P_F_)*^n^*.

“*It follows that a much larger BMR must be used in benchmark dose modeling for litters when inference about fetal risk is required and when the only data consists of the proportion of affected litters. The relation between P_F_ and P_L_ (Fig. 3) implies a corresponding relation of extra risk values for litters and offspring.*”

For the extra risk (*ER*) at dose *x*, $\log\left( 1-{ER}_{x} \right)=\log\left( 1-P_{x} \right)-log(1-P_{0})$. Thus, $\log\left( 1-{ER}_{x,L} \right)=b*\left[ \log\left( 1-P_{x,F} \right)-\log\left( 1-P_{0,F} \right) \right]= b*log \left( 1-{ER}_{x,F} \right)$.

There is a linear relation for *P_F_* < 0.05, ${log}_{10} \left( 1-P_{L} \right)=a+b*{log}_{10}(1-P_{F})$, where *a* = 0.001218, *b* = 9.559, estimated by orthogonal least squares. Note that 10^a^ = 1.002808 ≈ 1. Thus, to a good approximation, $(1-P_{L})={(1-P_{F})}^{b}$. This implies that the same value of b applies to $(1-{ER}_{L})={(1-{ER}_{F})}^{b}$ as derived just above. Using the estimate *b*≈ 9.6, fetal extra risk values of 0.01 and 0.05 correspond to per-litter extra risk values of 0.092 and 0.39. The relation between *P_L_* and *P_F_* is rather noisy, so it is best to use data on fetal proportions to estimate fetal risk.
