# Supplementary material for: Dose-Response Modeling with Summary Data from Developmental Toxicity Studies
Source: Risk Anal. Author manuscript; Available in PMC 2021 Aug 18. (PMC8372781; doi:10.1111/risa.12667)
Supplement: Supporting Information [file NIHMS1544377-supplement-Supporting_Information.zip › Regression of Deff on pF.docx]

**Regression of design effect (Y = *D*) on proportion malformed (X = *P*)**

The objective is to predict Y from X (Figure 1), preferably using a simple linear regression. The relation between log(Y) and log(X) is roughly linear (that between Y and X, not shown, is patently nonlinear).

Some problems with these data:

1. Y is calculated from X, so errors are correlated

2. Sampling error in X

3. Variance of X increases with X

There is no easy solution to these problems (McArdle 2003, Carroll and Ruppert 1996).

The easier solutions require knowledge of the errors. Less easy but still feasible solutions require use of instrumental variables (which are lacking in this case).

We observe W = X + e (e = error, with variance σ_ex_^2^). The estimated least-squares (LS) regression slope is COV[W,y] / VAR[W] while what is desired is COV[X,y] / VAR[X]. Error in X may reduce the estimated covariance of W and Y and may increase the variance of W.

In this case ("asymmetric relationship"), the recommended approach is to use a particular type of moment estimate (McArdle 2003, Carroll and Ruppert 1996). The corrected slope estimate is

β_MM_ = β_LS_ * { VAR[W] / ( VAR[W] - σ_ex_^2^ ) }.

This correction can be applied if σ_ex_^2^ is known. It can be estimated directly by replication (not possible in our case), or it can be estimated using an 'instrumental' variable correlated with X. Another possible way to estimate it is by jackknifing (see note below). However, this variance is not a constant - it increases with p (Figure 2). The variance of log(pF) is not known. It could be calculated from VAR[p] using a Taylor series approximation of dubious merit, but a better estimate would be desirable; after all this, it may not be a constant as assumed by the correction method above.

If the relation is linear, the correct estimate of the line will lie between the ordinary least squares (OLS) estimates for Y = a + bW and that for W = c + dY (after the latter is rearranged to the form Y = (c/d) + (1/d)W). These least-squares bounds are unhelpfully wide for our data (Figure 1), as often happens (McArdle 2003).

Least squares regression applied to this regression problem will under-estimate beta; orthogonal regression (OR) may, arguably, over-estimate beta in this case because sampling error for Y should be much larger than measurement or sampling error for X (McArdle 2003, Carroll and Ruppert 1996). The difference between these two bounds is small enough to be useful, as Figure 1 and tabulated results below will show.

For all the data (all species combined), the estimates for a linear relation between log_e_(D) and log_e_(P) are

|  | N | a | b | resid. var. σ_res_^2^ |
| --- | --- | --- | --- | --- |
| Ordinary Least Squares (OLS) | 232 | 1.366 | 0.2546 | 0.1790 |
| Orthogonal Regression (OR) | 232 | 1.465 | 0.2818 | 0.1656 |

Estimates of the mean of D may be calculated by transforming estimates back from log scale as exp( a + b*log(P) + 0.5* σ_res_^2^ ):

| P | 0.01 | 0.05 | 0.1 | 0.2 | 0.3 | 0.5 | 0.8 |
| --- | --- | --- | --- | --- | --- | --- | --- |
| LS | 1.12 | 1.67 | 1.99 | 2.38 | 2.64 | 3.00 | 3.39 |
| OR | 1.09 | 1.71 | 2.08 | 2.53 | 2.84 | 3.28 | 3.74 |

Examination of species-specific estimates suggests that different equations should be used for the three species (mice, rats, rabbits). Although the inferences are not exact for reasons noted above, rabbits differ significantly from the rodents, and all differ substantially in coefficient values.

| Linear least squares (LS) and orthogonal regression (OR) estimates by species, for the relation  log_e_(D) = a + b*log_e_(P), for cases with P>0. | | | | | |
| --- | --- | --- | --- | --- | --- |
|  |  | N | a | b | var. σ_res_^2^ |
| Mice | LS | 88 | 1.5938 | 0.2866 | 0.2078 |
| Mice | OR | 88 | 1.6943 | 0.3132 | 0.1863 |
| Rats | LS | 101 | 1.6852 | 0.3310 | 0.1248 |
| Rats | OR | 101 | 1.8327 | 0.3690 | 0.1090 |
| Rabbits | LS | 43 | 1.0582 | 0.2397 | 0.1452 |
| Rabbits | OR | 43 | 1.1477 | 0.2739 | 0.1299 |

| Estimated design effects by species for a range of proportions malformed (P) | | | | | | | |
| --- | --- | --- | --- | --- | --- | --- | --- |
| P: | 0.01 | 0.05 | 0.10 | 0.20 | 0.30 | 0.50 | 0.80 |
| mice LS | 1.46 | 2.31 | 2.82 | 3.44 | 3.87 | 4.48 | 5.12 |
| mice OR | 1.41 | 2.34 | 2.90 | 3.61 | 4.10 | 4.81 | 5.57 |
| rats LS | 1.25 | 2.13 | 2.68 | 3.37 | 3.85 | 4.56 | 5.33 |
| rats OR | 1.21 | 2.19 | 2.82 | 3.65 | 4.23 | 5.11 | 6.08 |
| rabbits LS | 1.03 | 1.51 | 1.78 | 2.11 | 2.32 | 2.62 | 2.94 |
| rabbits OR | 0.95 | 1.48 | 1.79 | 2.16 | 2.42 | 2.78 | 3.16 |

References

McArdle, B.H. (2003) Lines, models, and errors; regression in the field. Limnol. Oceanogr. 48:1363-1366.

RJ Carroll and D Ruppert (1996) The use and misuse of orthogonal regression in linear errors-in-variables models. The American Statistician 50:1-6

Reilman, M., R.F. Gunst and M.Y. Lakshminarayanan (1985) Structural model estimation with correlated measurement errors. Technical report No. SMU-DS-TR-192. 9 pages. http://www.smu.edu/~/media/Site/Dedman/Departments/Statistics/TechReports/TR-192.ashx?la=en

Figure 1. Design effect (*D*) versus proportion of malformed fetuses (*P_F_*) in the dose group, on log_10_ scale. There is one point for each dose group. Nine dose groups with *P_F_* = 0 are omitted. Species: mice, open circle; rats, filled circles; rabbits, asterisks. The lines show predictions based on a least squares regression (black, dashed) and an orthogonal regression (solid). The ‘inverse’ regression of log10(pF) on log10(D) is shown as a dashed line in red. The arc pattern at lower left is attributed to a lower limit to estimated design effect (related to sample size). Note the log base 10 scales.

Appendix I. Note on estimating measurement error using simulations.

Measurement error was explored by simulating 'developmental toxicity experiments', using a beta-binomial distribution for number of affected offspring to induce data clustering. Design effect was fixed at values 2, 4, and 6. The expected value of proportion affected was fixed at 0.01, 0.05, and 0.10. We used 20 litters and a mean of 15 offspring per litter. Number of offspring per litter was Poisson-distributed with a mean of 15 offspring per litter. Each 'developmental toxicity experiment' was repeated by simulation 1000 times. "Measurement error" in this simulation, as in reality, is identified with sampling error for *P* and for *D*.

| E[D] | E[P] | sample-var(D) | sample-var(P) | ratio (D/P)  sample var | correl * |
| --- | --- | --- | --- | --- | --- |
| 1.2 | 0.1 | 0.16 | 0.00037 | 419 | 0.18 |
| 2 | 0.1 | 0.63 | 0.00060 | 1050 | 0.26 |
| 4 | 0.1 | 2.7 | 0.0012 | 2250 | 0.39 |
| 6 | 0.1 | 6.7 | 0.0018 | 3722 | 0.45 |
| 1.2 | 0.05 | 0.18 | 0.00020 | 902 | 0.094 |
| 2 | 0.05 | 0.78 | 0.00032 | 2438 | 0.34 |
| 4 | 0.05 | 3.7 | 0.00068 | 5441 | 0.53 |
| 6 | 0.05 | 9.1 | 0.00099 | 9192 | 0.64 |
| 1.2 | 0.01 | 0.17 | 0.000039 | 4333 | 0.31 |
| 2 | 0.01 | 1.1 | 0.00007 | 15714 | 0.58 |
| 4 | 0.01 | 5.0 | 0.00014 | 35714 | 0.76 |
| 6 | 0.01 | 10. | 0.00020 | 50000 | 0.79 |
| * correlation of (D - E[D]) with (P - E[P]) | | | | | |

These simulations show that the measurement error for Y = D is two or three orders of magnitude greater than the error for X = P, and that correlation of errors is in the range 0.25 to 0.8.

To apply the results of Reilman et al. (1985), we need two parameters. Their parameter φ is the slope parameter scaled by the square root of the ratio of error variances of Y = D (σ_v_^2^) and X = P (σ_u_^2^): $\varphi=\beta\lambda^{-1/2}$ and $\lambda=\sigma_{v}^{2}/\sigma_{u}^{2}$ . Based on simulations above, λ^-1/2^ is on the order of 0.05 to 0.004, for D ≥ 2, and beta (estimated, not true) is approximately 0.2 to 0.3, so φ is probably less than 0.01.

We also need their parameter $\gamma=\sigma_{u}^{2}/\sigma_{X}^{2}$, the ratio of the error variance for X and the variance among actual X values. The latter is clearly small because the variance of measured X = P for our data is 0.0214 while the error variance for X based on simulations is probably no greater than 0.002 (see table above).

According to Figure 2 of Reilman et al. (1985), for this range of values for φ and γ, the OLS and ML estimators have very similar asymptotic mean squared errors for error correlations of 0 and 0.5. Thus there is no measureable loss to using the OLS estimate.

Appendix II. Note on jackknife estimate of VAR[pF]

Each observation of pF is a ratio estimate for one dose group. It is the ratio of the sum (over all litters) of the number of malformed fetuses to the sum (over all litters) of the number of all fetuses. This estimate has two components of variance, that among fetuses within litters and that among litters (which may differ intrinsically in expected proportion of malformations). The variance among fetuses within a litter might be a binomial variance, but even then this variance will differ among litters to the extent that litters differ in expected proportions.

The traditional sample estimator of VAR[pF] is given by Rao and Scott (1992) and is based on a 2nd-order approximation. This was estimated for the sample data. On a log-log scale, sqrt(VAR[pF]) is linear in pF (Figure 2).

Another possible way to estimate the sampling variance of pF is by jackknifing the data for a dose group. This would need to take into account the hierarchical structure (fetuses within litters). For a bootstrap estimate, it is known that resampling of whole litters is more accurate than resampling both within and among litters (AC Davison and DV Hinkley 1997 Bootstrap Methods and Their Application).

Figure 2. Relation of estimated standard deviation of pF, the ratio estimate of proportion malformed in a dose group, to that proportion pF, on a log scale.

**Simulation code**

sim.litters.Deff.fn <- function(nsim=1, nlitters=20, npups=15, ep=0.10, D=2,

littersize.distrib = c("poisson", "negbinom", "constant")[1], mu=npups,

scale.nb = 1)

{

# #####################################

# local function 'vp()'

vp <- function(y){

m <- dim(y)[1] # nlitters

x <- y[,1]

n <- y[,2]

x0 =0; xn = 0

# estimates of ep and Vp (Rao-Scott):

phat <- sum(x)/sum(n)

vp <- (m/(m-1))*(sum(n)^-2)*sum( (x - n*phat)^2 )

if(all(x == 0)) x0 = x0 + 1

if(all(x == n)) xn = xn + 1

Deff = sum(n)*vp/(phat*(1-phat))

# litter-means pi and their variance

pi = x/n

mn.pi = mean(pi)

var.pi = var(pi)

#bvpi = mpi*(1-mpi)/m

mn.lsz = mean(n)

c(Deff=Deff, phat=phat, vp=vp, mn.pi=mn.pi, var.pi=var.pi, mn.lsz=mn.lsz, x0=x0,xN=xn)

}

# #####################################

#########################################

# START

# set up beta distrib: ep = a/(a+b); b = a*((1-ep)/ep);

a.beta = ep * ( (npups-1)/(D-1) -1)

b.beta = a.beta*(1-ep)/ep

# set up number of pups per litter

n.i <- switch( littersize.distrib,

poisson = rpois(n = nsim*nlitters, lambda = npups),

negbinom = rnbinom(n = nsim*nlitters, mu = npups, size = (mu/scale.nb)),

constant = rep(npups, nsim*nlitters)

)

# generate pi.i (unobservable) for each dam (litter)

p.i <- rbeta(n = nsim*nlitters, shape1=a.beta, shape2=b.beta)

# generate samples

x.i <- matrix( rbinom(n = nsim*nlitters, prob = p.i, size = n.i), ncol=nsim )

n.i <- matrix( n.i, ncol=nsim)

# dim x.i, n.i is c(nlitters, nsim)

# get sample statistics

xs <- apply(x.i, 2, sum) # sum of affected pups over nlitters, length nsim

ns <- apply(n.i, 2, sum) # sum of total pups over nlitters, length nsim

#

# sample estimate of ep, length nsim:

pxhat <- xs/ns

#

# get Rao-Scott Variance estimator (call function 'vp'), etc.

xn = array( c(x.i,n.i), dim=c(nlitters, nsim, 2))

Vp <- apply(xn, 2, vp)

# returns c(Deff=Deff, phat=phat, vp=vp, mpi=mpi, vpi=vpi, x0, xN)

# dim(Vp) = c(8, nsim)

Mdeff = mean(Vp[1,], na.rm=T)

Vdeff = var(Vp[1,], na.rm=T)

print(paste("nsim =", nsim, ", nlitters =", nlitters, ", npups =", npups, ", E[p] =", ep, ", DEFF =", D, ", littersize.distrib =", littersize.distrib))

print(paste("no.sim.all.x=0", sum(Vp[7,]), "no.sim.all.x=N", sum(Vp[8,]) ))

print(paste("Mean(pi) =", round(mean(Vp[4,]),4), ", Var(pi) = ", round(var(Vp[4,], na.rm=T),6), ", no.na =", length(Vp[4,][!is.finite(Vp[4,])]),

", corr.err(D,p) =", round(cor( (Vp[1,] - D), (Vp[4,] - ep), use="na.or.complete"),6) ))

print(paste("Parametric Deff = ", D, ", sample mean Deff = ", round(mean(Vp[1,], na.rm=T),3), ", sample var Deff = ", round(var(Vp[1,], na.rm=T),3) ))

#########################################

# wrap up and report

invisible(Vp)

} # END

#######################################################################

# > sim.litters.Deff.fn(nsim=1000, ep=0.1, D=2)

# [1] "nsim = 1000 , nlitters = 20 , npups = 15 , E[p] = 0.1 , DEFF = 2 , littersize.distrib = poisson"

# [1] "no.sim.all.x=0 0 no.sim.all.x=N 0"

# [1] "Mean(pi) = 0.1011 , Var(pi) = 0.000596 , no.na = 0 , corr(D,p) = 0.2632"

# [1] "Parametric Deff = 2 , sample mean Deff = 2.061 , sample var Deff = 0.631"

#

# > sim.litters.Deff.fn(nsim=1000, ep=0.1, D=4)

# [1] "nsim = 1000 , nlitters = 20 , npups = 15 , E[p] = 0.1 , DEFF = 4 , littersize.distrib = poisson"

# [1] "no.sim.all.x=0 0 no.sim.all.x=N 0"

# [1] "Mean(pi) = 0.1002 , Var(pi) = 0.001203 , no.na = 0 , corr(D,p) = 0.390929"

# [1] "Parametric Deff = 4 , sample mean Deff = 3.984 , sample var Deff = 2.71"

#

# > sim.litters.Deff.fn(nsim=1000, ep=0.1, D=6)

# [1] "nsim = 1000 , nlitters = 20 , npups = 15 , E[p] = 0.1 , DEFF = 6 , littersize.distrib = poisson"

# [1] "no.sim.all.x=0 0 no.sim.all.x=N 0"

# [1] "Mean(pi) = 0.0981 , Var(pi) = 0.001757 , no.na = 0 , corr(D,p) = 0.449732"

# [1] "Parametric Deff = 6 , sample mean Deff = 5.922 , sample var Deff = 6.695"

#

# sample var of "D" is two orders of magnitude (>100x) greater than that of "p"

#######################################################################

# > sim.litters.Deff.fn(nsim=1000, ep=0.05, D=2)

# [1] "nsim = 1000 , nlitters = 20 , npups = 15 , E[p] = 0.05 , DEFF = 2 , littersize.distrib = poisson"

# [1] "no.sim.all.x=0 0 no.sim.all.x=N 0"

# [1] "Mean(pi) = 0.0501 , Var(pi) = 0.000321 , no.na = 0 , corr(D,p) = 0.340836"

# [1] "Parametric Deff = 2 , sample mean Deff = 1.983 , sample var Deff = 0.777"

#

# > sim.litters.Deff.fn(nsim=1000, ep=0.05, D=4)

# [1] "nsim = 1000 , nlitters = 20 , npups = 15 , E[p] = 0.05 , DEFF = 4 , littersize.distrib = poisson"

# [1] "no.sim.all.x=0 0 no.sim.all.x=N 0"

# [1] "Mean(pi) = 0.0509 , Var(pi) = 0.000679 , no.na = 0 , corr(D,p) = 0.528397"

# [1] "Parametric Deff = 4 , sample mean Deff = 3.591 , sample var Deff = 3.732"

#

# > sim.litters.Deff.fn(nsim=1000, ep=0.05, D=6)

# [1] "nsim = 1000 , nlitters = 20 , npups = 15 , E[p] = 0.05 , DEFF = 6 , littersize.distrib = poisson"

# [1] "no.sim.all.x=0 17 no.sim.all.x=N 0"

# [1] "Mean(pi) = 0.049 , Var(pi) = 0.000988 , no.na = 0 , corr(D,p) = 0.638781"

# [1] "Parametric Deff = 6 , sample mean Deff = 5.023 , sample var Deff = 9.141"

#

# sample var of "D" is two orders of magnitude (>100x) greater than that of "p"

#######################################################################

# > sim.litters.Deff.fn(nsim=1000, D=2, ep=0.01)

# [1] "nsim = 1000 , nlitters = 20 , npups = 15 , E[p] = 0.01 , DEFF = 2 , littersize.distrib = poisson"

# [1] "no.sim.all.x=0 129 no.sim.all.x=N 0"

# [1] "Mean(pi) = 0.0104 , Var(pi) = 7e-05 , no.na = 0 , corr(D,p) = 0.584289"

# [1] "Parametric Deff = 2 , sample mean Deff = 1.69 , sample var Deff = 1.049"

#

# > sim.litters.Deff.fn(nsim=1000, D=4, ep=0.01)

# [1] "nsim = 1000 , nlitters = 20 , npups = 15 , E[p] = 0.01 , DEFF = 4 , littersize.distrib = poisson"

# [1] "no.sim.all.x=0 308 no.sim.all.x=N 0"

# [1] "Mean(pi) = 0.0098 , Var(pi) = 0.00014 , no.na = 0 , corr(D,p) = 0.76228"

# [1] "Parametric Deff = 4 , sample mean Deff = 2.767 , sample var Deff = 5.002"

#

# > sim.litters.Deff.fn(nsim=1000, D=6, ep=0.01)

# [1] "nsim = 1000 , nlitters = 20 , npups = 15 , E[p] = 0.01 , DEFF = 6 , littersize.distrib = poisson"

# [1] "no.sim.all.x=0 414 no.sim.all.x=N 0"

# [1] "Mean(pi) = 0.01 , Var(pi) = 0.000204 , no.na = 0 , corr(D,p) = 0.78824"

# [1] "Parametric Deff = 6 , sample mean Deff = 3.847 , sample var Deff = 10.14"

#

#######################################################################
